# Tuberculosis and the French Congress for Its Study

**Published:** 1893-11

**Authors:** R. H. L. Bibb

**Affiliations:** Saltillo, Mexico


### TEXAS MEDICAL JOURNAL.

ESTABLISHED JULY, 1885.

PUBLISHED MONTHLY.—SUBSCRIPTION $2.00 A YEAR.

VOL. IX AUSTIN, NOVEMBER, 1893. NO. 5.

### Original Contributions.

For Texas Medical Journal.

### TUBERCULOSIS AND THE FRENCH CONGRESS FOR ITS STUDY.

R. H. L. BIBB, M. D., SALTILLO, MEXICO.

IN VIEW of the great importance of everything tending to a better understanding of the nature, prevention, and cure, of tuberculosis, it is believed that a short review of ideas expressed, and experiences related, at the recent meeting of the French Congress for the study of tuberculosis in Paris, July 27 to August 3, of this year, by a few of France's wisest, most scientific, and experienced physicians, will not be wanting in interest to those to whom such information must needs come indirectly.*

Concerning the respective parts played by inheritance and contagion, in the diffusion of tuberculosis among animals, M. Nocard, although not absolutely denying its transmission from parent to offspring, regarded it as of secondary importance in comparison with contagion, which latter must be intimate and prolonged to affect healthy animals. M. Empis, who had never seen a case of contagion in man, and who doubted its occurrence even between husband and wife, declared the opposite true in the human race—inheritance being its chief, if not only, means

* The data on which this article is based is taken from "La Clinique Francaise" for August, 1893.

of dissemination. To this assertion M. Hèrard responded that the fact of contagion, especially between husband and wife, was, and had often been, too incontestably proven to admit of even a shadow of doubt.

M. d'Hôtel (Poix-Terron, Ardennes), speaking from an experience gained in fifty years of active practice among rural populations, where it was easy to observe families for several generations, where he could follow up cases of contagion, and where he had studied tuberculosis in the light of heredity and contagion in a series of fifty observations, containing from two to twelve patients, each, concludes the disease is not hereditary in the absolute meaning of the term; that it transmits, by inheritance, a predisposition, and that it increases in contagiousness with density of population.

In 75 cases of infantile—surgical—tuberculosis, observed by M. Goudry, only ten cases were hereditary. He believes instances of inherited form of the disease extremely rare, that from contact alarmingly frequent.

Speaking of Belgium, M. Degire declared that tuberculosis, by contagion, was a veritable plague among the peasantry of that country, and that, principally as a contagious disease, did it merit notice from a sanitary standpoint.

In 17 animals, inoculated with tuberculine by M. Siegen (Luxemburg), for diagnostic purposes, ten showed marked reaction, and, on being killed, were found to be tuberculous, while of the seven, showing no reaction, no evidence of disease could be found. According to this observer, the most insignificant deposit reacts to tuberculine; but the amount of deposit does not influence the degree of reaction.

Of 410 animals, inoculated with tuberculine by M. Degire, 183 reacted, and were demonstrated to be diseased. He considers the reaction pathognomonic of tuberculosis, and believes the amount of reaction is determined by the extent of the disease.

M. Aubeau (Paris), honorary president of the Clinical Society of Physicians of France, announced that by bacteriological examinations of the blood, milk, spermatic, and other fluids, he was able, not only to diagnose tuberculosis in advance of the usual symptoms and signs relied on for that purpose, but could diagnose the disease by this method, while yet none other would furnish the faintest suspicion of its presence. He stains his preparations with Ziehl's carbol-fuschin, and exhibited various specimens of milk, blood, and spermatic fluid, prepared with the assistance of M. Galasz, containing Koch's bacillus. M. Aubeau believes his discovery explains and proves the direct transmission of the *bacillus tuberculosis* from father to child—an infection at the moment of fecundation.

Various members of the Congress—M. M. Verneuil, Nocard, Strauss and others—argued the possibility of the patient furnishing the spermatic fluid containing tubercle bacilli, having an inappreciable tuberculosis of the testicles, or of the seminal ducts, M. Strauss, basing his opinion on the fact that the bacilli in the specimen, were not isolated, but grouped in bunches, as in sputum.

Replying, M. Aubeau denied that the patient had tuberculosis of any of the genital organs, and stated he had other analogous preparations. He asserted that in spermatic fluid from cases of genital tuberculosis, the bacilli are found in and among the leucocytes, mucus, pigment, spermatozoids, and cellular debris, while in cases without appreciable genital involvement, nothing was encountered in the spermatic fluid but the bacilli and spermatozoa; and that in the preparation submitted to the Congress, not a leucocyte, fragment, or fiber of any kind, was to be seen; on the contrary, it was remarkable for its purity, and the absence of everything else but the spermatozoa, colored blue, and the bacilli, colored red. M. Aubeau reiterated the assertion that he had often detected tubercle bacilli in milk, human and animal, when no other evidence of the disease could be disclosed, in the mammary glands, or in the patients themselves.

M. Degire added that, inasmuch as Koch's bacillus had, undoubtedly, been frequently found in milk from tuberculous cows with healthy udders, it was quite rational to expect their presence in spermatic fluid from sound genital organs.

M. Nocard insisted that in all such cases there certainly existed in the lactiferous and in the genital organs, foci of infection which had been overlooked, possibly, because of their extreme minuteness.

Admitting the validity of objections urged by Verneuil, Nocard and Strauss, it is claimed that the importance of any method that can be applied to the milk and spermatic fluid for determining the presence of tuberculosis in the milk and generative organs not appreciable to other methods of diagnosis, cannot be overestimated. Hence it is that the admirers of M. Aubeau insist that objections, possibilities and probabilities, even from such eminent sources, are powerless to detract from the importance of his discovery.*

M. Babés, who, in conjunction with M. Cornil and Deloir, has been experimenting since 1883 with vaccinations of attenuated tubercular virus from rabbits; while Koch and Grancher were experimenting with cultivated virus attenuated by age, and while Richet and Hericourt were demonstrating that dogs could be made refactory to human tuberculosis by injections of cultivated aviarian virus, related his experience in producing immunity in animals and in the use of immunized serum (serum-therapy) in animals and in man.

He stated he had produced absolute immunity in dogs, immunity to injections of large quantities of human virus, by commencing with injections of considerable amounts of aviarian virus, repeating in a few days and periodically, the inoculation quantities of virulent cultivations. His process is:

1. An injection of aviarian tuberculine.

2. An injection of a cultivation of aviarian virus attenuated by one year's age.

3. In eight days an injection of one gram of a one month's cultivation.

4. Eight days after, an injection of three grams of the same cultivation.

5. Eight days after, five grams of the same cultivation.

6. Human tuberculine.

7. Old human cultivation.

8. Five centigrams of recent human cultivation.

9. Twenty days after, one gram of the same cultivation.

10. In ten days, another injection of two grams of the same cultivation, and so on, until the animal is completely immunized.

The process of immunization was very fatal to the animals experimented with. Out of 20 dogs, 50 rabbits and 2 guinea pigs, used in these experiments, M. Babés had left, at the end of the year, and immunized, 4 dogs, 2 rabbits and 1 guinea pig,

---

*Commenting on the importance of this discovery, the editor of "Le Medicine Practique" writes: "If we have such a diagnostic element, while nothing else appears to reveal tubercular activity; if we have such precious means of enlightenment, then we have something destined to revolutionize the prophylaxis and hygiene of tuberculosis * * * the possibility of killing the bacillus before it begins its terrible work of destruction, can be onsidered.

the others having died from parenchymatous nephritis due, as he thinks, to associated microbes, Many of his animals died after considerable immunity had been produced in them.

M. Babés stated that he had used the serum of the blood of dogs thus immunized, in the treatment of tuberculosis (human and animal) and tubercular leprosy, giving from 3 to 6 grs., with one per cent of carbolic acid, hypodermatically, daily, with "marked benefit," "ab initio," in every grade of tuberculosis and tubercular leprosy. All of his patients increased in weight, strength and appetite. Cavities contracted, cough and expectoration diminished, and in two cases, the bacilli disappeared completely from the sputum. In leprosy, while the lepromas lessen in size and the patient showed other evidences of improvement, serum therapy is less potent than in tuberculosis.

Inasmuch as its vaccinæ, as demonstrated in his experiments, are greater than its therapeutical properties, M. Babés proposes to vaccinate children from tubercular parents with immunized serum in order to fortify them against and protect them from, the dreadful disease that threatens them.

M. Bernheim (Paris) has made many efforts to immunize animals, using cultivations attenuated by age, broths rich in Koch's bacilli destroyed by heat, and cultivations attenuated with antiseptics, but only accomplished his purpose after resorting to a method analogous to that of Behring, Kitaseta and others for diphtheria and tetanus. He heats for one hour and a half, to 80° Cent., a pure culture of tubercule bacilli, which he passes, at once, through a Chamberland filter. Of this filtrate, he injects ("intra-vascular") about twenty drops for each twelve pounds of the animal's weight. Animals subjected to this process, although repeatedly inoculated with virulent cultures of tubercle bacilli, have been found completely refactory. In some cases, however, when the injections had been made with a highly concentrated virus, some of the animals died of ante-intoxication associated with non-tubercular abscesses.

With serum obtained from the blood of animals immunized in this manner, Bernheim injected other animals, first, immediately before; second, immediately after being inoculated with a higher concentrated and virulent virus, and, third, animals with extensive tubercular lesions, with the result that in no case, in the first and second series, has an animal died of tuberculosis, while those of the third series died in great numbers; but much later than did control animals of the same series. In the animals of the third series that did not succumb to tuberculosis and were afterwards killed, autopsies showed the tubercular lesions to be in process of active repair.

Bernheim stated that he had used immunized serum in 105 cases of human tuberculosis in every phase and grade of the disease, and with, invariably, favorable results. Those without advanced or grave symptoms have been cured, temporarily, at least, inasmuch as the bacilli, and all physical, objective and subjective signs have disappeared.

He regards this serum-therapy as absolutely harmless, and recommends that from one to two hundred drops (5 to 10 c. c.) of it be injected in the interscapular region every second day until forty injections have been made, by which time its effects, favorable or otherwise, will have been demonstrated.

In Bernheim's opinion, the favorable results following the use of immunized serum are neither due to tonic action nor to psychic suggestions, but to a real, a specific vaccinal property against the bacillus tuberculosa and its toxines, by which the latter are neutralized, thus permitting leucocytic and phagocytic action, and in turn, destruction of the bacilli themselves. It being impossible, in his conception, to saturate the organism with medicaments sufficiently active to destroy the bacilli without endangering the life of the individual, Bernheim declared it was high time for the profession to abandon the false idea of direct attack upon these micro-organism, in favor of the safe and powerful process offered by immunized serum.

During his investigations, Bernheim treated numbers of consumptives with artificial and with ordinary serum, and with antiseptic medications,—methods of undoubted utility, but, as demonstrated by him, greatly inferior to immunized serum, as evidenced by the fact that patients with rapid, febrile phthisis, not affected by injections of phenicated oils, nor when saturated with the salicylates, the safest, best and most efficacious of all the anti-microbics against the tubercle bacillus, have invariably improved, and at once, on changing to immunized serum, many having been cured.

While not a believer in direct transmission of tuberculosis from parent to offspring, Bernheim recognizes in such subjects a constitutional vice that renders them peculiarly susceptible to the tubercle bacillus, and joins M. Babés in urging immunized serum as a protective measure in such cases.

M. Ch. Leroux (Paris) reported that in fifty cases of infantile pulmonary tuberculosis treated with hypodermic injections of iodoform at the Furtado-Heine dispensatory, thirty-five showed various degrees of improvement, local and general, while in the remainder, many of whom were injured, no good results were obtained.

M. de la Jarrige injects, daily, into the tracheas of his phthisical patients, with varying results, from one to two ounces of the following mixture: Menthol, 70 grains; creosote, 2½ drams; sterilized oil, 4 ounces, and claims that it is well tolerated and quickly absorbed.

Weil and Diamantberger use equal parts of gayicol and oil of sweet almonds, and inject it, subcutaneously, in from one to twelve drams doses, every twenty-four hours, with, as they assert, benefit and safety.

Microscopists and pathologists agree that tuberculosis is a parasitic disease, and there is great unanimity among clinicians that it is disseminated chiefly by infection or contagion. According to statisticians, the disease is alarmingly on the increase, while the experience of most therapeutists is, that it is almost, if not quite, incurable. Looking at the subject in the light of these facts, for facts they are, it seems pertinent to inquire: What are the exigencies of the hour, how are they to be met, and by whom? A proper response to these inquiries, one that will fill the requirements, is too broad, too intricate and too important to be attempted in this paper, did ability permit or space admit the report.

Whatever may be the final solution of these all-important problems, whether by vaccination with immunized serum as proposed by Babés, Bernheim, and others, or whether by invocation "of the strong arm of the law," as is being seriously discussed in some parts of Europe, and the victims of tuberculosis isolated, as in cholera, yellow fever, etc., much may be done, even now, by physician and patient towards preventing the spread of consumption, by strictly observing the suggestions of Dr. Rochester, published in the *Medical News* of September 2, 1893. The "Pennsylvania society for the prevention of tuberculosis" offers, through its secretary, E. Leslie Gilliams, 806 Walnut street, Philadelphia, to supply its circulars, "*How to avoid contracting tuberculosis*," and "*How persons suffering from tuberculosis can avoid giving the disease to others*," to all who will join the society and pay one dollar, in such numbers as they can advantageously use, without other expense. The circulars will be furnished any one, however, whether a member or not, on application to the secretary. Physicians, everywhere, should exercise themselves in this behalf, and see to it, that these valuable little publications are scattered broadcast throughout the land.

On the dangers arising from dried tuberculosis sputum, an editorial, on "The prevention of phthisis," in the *British Medical Journal* of September 23, 1893, has this to say: "All evidence goes to show that putting on one side infection by food, which comes under a different branch of sanitary work, the great and constantly acting cause of phthisis is the respiration of phthisically infected dust, and that the source from which the dust becomes infected is the sputum of consumptive patients. Whether we look on the large incidence of phthisis on those who pass their time in dusty workshop, and who dwell in dirty, ill-kept houses, as proof of the direct infectiousness of the disease, or whether we accept the view, held by some, of the saprophytic existence of the bacillus tuberculosis—that is, of its power to develop apart from the living body, to live and grow on damp walls in dark dwellings, as distinct from its merely resting there as a spore, and thus make the disease become endemic in 'tubercular infective areas,'—in either case we must look to dust as the means of communicating the infection, and to want of cleanliness as the cause of its continuance. We wish we could feel sure that the medical profession at large recognize, as fully as the facts warrant them in doing, the infectiousness of tuberculous sputum and the danger of letting it dry into dust."

---

For Texas Medical Journal.

